# An Overview on Formation of Radiation-Induced Interface Traps in Silicon-Based Devices

**DOI:** 10.3390/mi16111278

**Published:** 2025-11-13

**Authors:** Xuehui Dai, Min Zhu, Fei Wu, Yanru Ren, Minghui Liu

**Affiliations:** Naval University of Engineering, Wuhan 430033, China

**Keywords:** ionizing radiation, interface trap, formation, properties

## Abstract

In an ionizing radiation environment, the formation of interface traps affects transistor performance, which may lead to device failure. This article reviews interface trap formation mechanisms in silicon-based devices. It explores interface trap types, electrical properties, and their impacts on devices’ performance. Finally, the main factors affecting the formation of interface traps are summarized. By reviewing these issues and exploring future research directions, guidance will be provided for the design of radiation-resistant devices to enhance their reliability in irradiated environments.

## 1. Introduction

Since ELDRS (enhanced low dose rate sensitivity) was first discovered in bipolar linear transistors (1991), researchers have carried out ionization radiation damage experiments on various devices with different structures, of which several models have been proposed [1,2,3,4]. Over the years, the mechanisms and basic characteristics of the effects of ionizing radiation damage on bipolar devices and MOS devices have always been hot-button issues of concern. Especially in extreme environments, such as those present in aerospace electronics, nuclear industry, and high-energy physics, the reliability of devices exposed to low-dose-rate radiation for a long time has attracted much attention. In early studies, researchers recognized that there are two main defects in ionizing radiation damage of devices: oxide-trapped charges and interface traps [5]. Some studies have shown that the gain attenuation of lateral PNP transistors is mainly dominated by interface traps [6]; in NPN transistors, both interface traps and oxide charges reduce the current gain [7]. Therefore, it is very important to clarify the formation mechanism of interface traps.

In general, interface traps refer to defect states existing at the interface between a semiconductor and an insulator (such as silicon dioxide) or between two non-insulating materials. These defect states can capture carriers (electrons or holes), which in turn affects the performance of the device. Research on interface traps induced by low-dose-rate radiation is an important topic in the field of radiation effects and semiconductor device reliability. As research progresses, a large number of experimental tests, mechanism studies, and models have been conducted on topics which include trap structure, formation process, and influence of different environments on interface traps. The significance of interface traps on device performance has gradually been recognized. This paper mainly summarizes the formation and electrical properties of interface traps in silicon-based devices, providing guidance for device process improvement and enhancing device performance and reliability.

## 2. Formation Mechanisms of Interface Traps

### 2.1. Hole Trapping and Recombination Model

Under a positive gate bias, holes generated by ionizing radiation will drift towards the SiO_2_/Si interface. Some of these holes are trapped, then the trapped holes are transformed into interface trap defects through specific reactions. Therefore, early experts believed that trapped holes were the cause of interface defects [8,9,10].

In 1980, research by Hu et al. [11] showed that, for MOS capacitors with dry-grown oxides under a field stress of 7.1–7.5 MV/cm and a temperature of 90 K, the accumulation of trapped holes occurred. The number of interface states generated within 1 h at 20 or 66 °C was linearly proportional to the number of trapped holes; when the capacitors were stored at room temperature for 1 year, the number of interface states generated in the 0.7 eV part at the center of the Si bandgap was essentially equal to the original number of trapped holes. These results provide quantitative evidence of the relationship between trapped holes and interface states.

**Figure 1:**
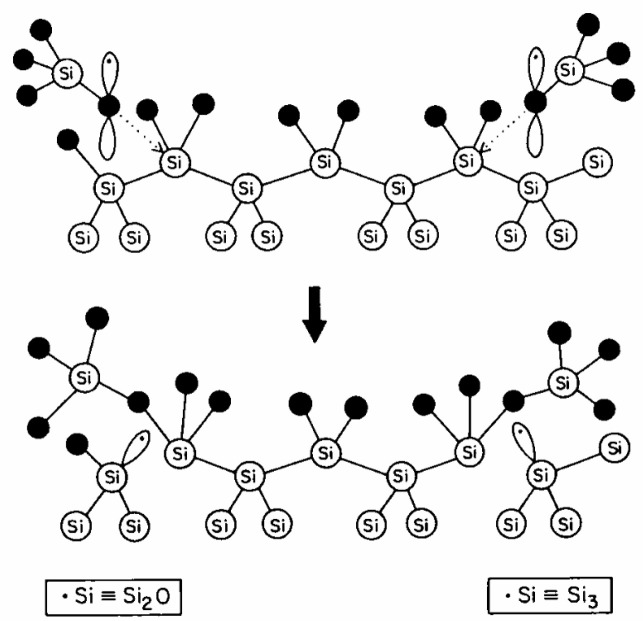
The formation of interface states at the SiO_2_/Si interface upon termination of the migration of the non-bridging oxygen [14].

Subsequent researchers continued to conduct model studies at the mechanistic level. Between 1980 and 1986, Grunthaner and others from the California Institute of Technology conducted XPS (X-ray photoelectron spectroscopy) studies on the structure, effects, etc., of trapped holes. They analyzed the chemical and electrical structures of the SiO_2_/Si interface and proposed the BSG (Bond Strain Gradient) model [12,13,14]. This model assumes that holes induced by radiation are trapped in the narrow strain transition layer of the oxide near the Si interface. This process breaks a strained Si-O-Si bond, generating an immobile trivalent Si (•Si≡Si_3_) and a mobile non-bridging oxygen (•Si≡Si_2_O). The non-bridging oxygen propagates towards the interface; an unspecified reaction occurs after it reaches the interface, which then gives rise to interface states [14], as shown in Figure 1:

Early studies generally held that interface states were caused by the transport of holes generated by radiation to the SiO_2_/Si interface. This transport led to the bonds breaking and subsequent increase in interface state density. In wet oxides, this increase was a slow process that occurred under positive bias and might be caused by the field-induced diffusion of ions out of the interface region [9]. In dry oxides, the broken bonds were likely to be strained bonds, and new interface states emerged immediately after the holes arrived. Furthermore, if they persisted for a long time, a positive electric field had to be maintained at this interface. Under a negative gate bias, little or no growth in the density of interface traps was observed [10].

The second viewpoint holds that the electron–hole recombination may cause some interface defect generation. When holes are trapped near the SiO_2_/Si interface and electrons are subsequently injected, electron–hole recombination occurs. The energy released through electron–hole recombination can lead to the formation of interface states. In 1983, Lai [15] from the IBM Thomas J. Watson Research Center discovered that by recombining electrons at the trapped holes, local defects will appear in the bandgap, and the density of these defects increases linearly with the number of recombination events.

There are two types of trapped holes near the Si/SiO_2_ interface; they are different in positions and electron capturing behaviors [16]. “Near-interface trapped holes” are located between 20 and 70 $\overset{\circ }{A}$ away from the interface; these holes completely disappear after capturing electrons. “Interface trapped holes” are located within about 18 $\overset{\circ }{A}$ of the interface; these holes immediately become interface states upon capturing electrons. Subsequently, Wang et al. [17] studied how these holes in the oxide of MOS capacitors evolved into interface states; they discovered the relationship between the position of trapped holes and the subsequent generation of interface states. First, holes are trapped between 20 and 70 $\overset{\circ }{A}$ away from the Si/SiO_2_ interface and then transferred within 18 $\overset{\circ }{A}$ of the interface; second, the holes capture electrons and transform into interface states.

In 1995, DiMaria et al. [18] discovered that t trapped holes can induce “slow” interface states, which are distinctly different from other interface states. These slow states can be generated and removed through successive hole generation and annihilation; these slow interface states depend on their distance to the SiO_2_/Si interface.

### 2.2. Hydrogen Models

For most fabrication and packaging technologies, hydrogen is used to ensure devices’ performance. On the other hand, hydrogen has been considered as a direct cause in degrading bipolar device TID response [19]. In hydrogen models, hydrogen exists in several forms, maybe neutral hydrogen atoms or hydrogen molecules. They can diffuse in oxide, react with oxygen vacancy defects or cracks at specific sites; this reaction will release a proton, then finally forms interface traps.

#### 2.2.1. Two-Stage Model

In 1978, Svensson [20] proposed a model for the defect structure at the Si-SiO_2_ interface. This model is based on the assumption of three forms of trivalent silicon in the Si-SiO_2_ interface; trivalent silicon and its hydrogen compounds are the most important defects in the Si-SiO_2_ system. When the hole trap captures the oxide hole, a reaction occurs to generate interstitial hydrogen (Hi), and excessive Hi will produce interface traps.

In 1979, Winokur et al. [21] conducted experiments and analyzed the formation of interface states in wet oxide MOS capacitors under a positive bias. The results showed that the long-term accumulation process of interface states needs to be described in two stages. The first stage occurs within the time required for holes to be transported to the SiO_2_/Si interface (<1 s). This stage determines the final value or saturation value of the interface states, and it was found that this stage is related to the electric field but independent of temperature. The second stage starts after the holes reach the SiO_2_/Si interface; it lasts for thousands of seconds. It determines the time scale of the formation of interface states, which is related to both the electric field and temperature.

Based on Winokur’s research, McLean proposed an empirical model [22] that explained the time-dependence of the accumulation of interface states. It can be regarded as the most complete two-stage model proposed at that time. Holes generated by radiation release hydrogen ions in SiO_2_ and pass through the oxide (the first stage). In the second stage, hydrogen ions undergo dispersive hopping transport, and further interactions occur after they reach the interface. The two-stage characteristics can be mathematically expressed as the following equations:

∆N_ss_(t) = N^∞^_ss_ (E_1_,D) f(t/τ) (1)

Τ = τ_0_ exp(∆/ΚT − αE_2_) (2)

where N_ss_(t) denotes the change in interface state density as a function of time; N^∞^_ss_ (E1,D) is the saturated values of N_ss_, it’s a function of dose D and the field E_1_ applied during the first stage; f(t/τ) is the second stage function, τ dependes on the temperature T and field E_2_ applied during the long-term buildup stage.

In addition, this study used field switching experiments to demonstrate the existence of an ion release mechanism in the first stage, and the most likely candidate for the released positive ions is H^+^. A portion of the protons are transported to the Si/SiO_2_ interface and react with the hydrogen-passivated silicon dangling bonds to form interface traps.

In 1988, the research by Griscom et al. [23] suggested that in the first stage, H^+^ was formed through the following reaction, where h^+^ denotes a hole. This process relied on release of H in the oxide and it was believed that H was released in the form of neutral molecular hydrogen (H_2_).

h^+^ + H(in oxide) → H^+^ (3)

**Figure 2:**
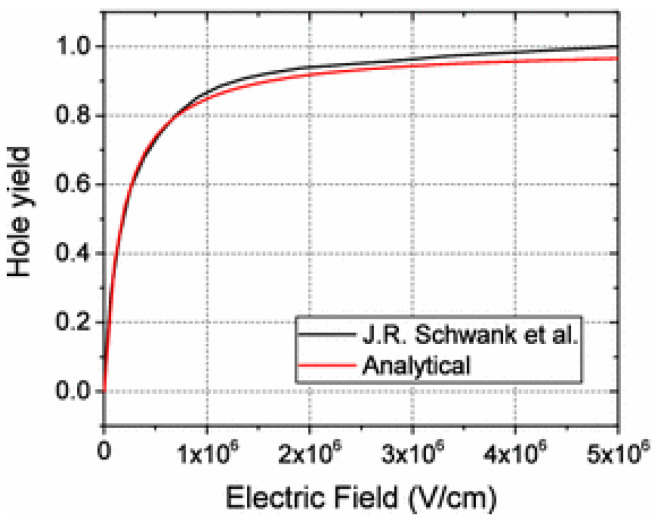
Hole yield as a function of electric field [25,26].

In 1989, Saks and Brown [24] developed a model to explain the generation of H^+^ in oxides. In the first stage, holes generated by radiation react with hydrogens in oxide to form H^+^. Through experiments and research, it is observed that radiation uniformly generates holes in the oxide (Figure 2) [25], and holes move along the direction of the electric field. The process of H^+^ formation is as follows: H^0^ (high-activity neutral hydrogen) is generated due to the recombination of radiation-induced electron–hole pairs, and then H^0^ reacts with another hole to form H^+^.

In 1990, Shaneyfelt et al. [27] proposed the (HT)^2^ (Hole Trapping/Hydrogen Transport) model, which explained the radiation-induced oxide and interface trap charge accumulation in polysilicon and metal gate transistors within a wide electric field range. In this model, under a positive bias during irradiation, holes are transported to the Si/SiO_2_ interface and trapped at 10 nm within Si/SiO_2_ interface. During the trapping process, H^+^ is released. Then, H^+^ is transported to Si/SiO_2_ interface and forms an interface trap through specific reactions. Under a negative bias, hole trapping near the gate/SiO_2_ interface can similarly release H^+^, then drift to the Si/SiO_2_ interface and form interface traps during the subsequent positive bias annealing period. In the first stage, protons are generated through the following reactions (Equations (4)–(6)).

D_B_H + p → D_B_H^+^ (4)

D_B_H^+^ → D_B_ + H^+^ (5)

D_B_H^+^ + n → D_B_H (6)

where p denotes a hole, D_B_H is Hydrogenated E’ center, D_B_H^+^ is positively charged Hydrogenated E’ center.

In the second stage, protons and passivated P_b_ centers (P_b_H) react via the reaction below (Equation (5)), which produces H_2_ and a dangling bond P_b_^+^, i.e., interface trap.

P_b_H + H^+^ → P_b_^+^+H_2_ (7)

Rashkeev et al. [28,29] conducted first-principle calculations and determined that H^+^ is the only stable charge state on the interface. H^+^ can react with Si-H to form H_2_ molecule and a positive dangling bond (i.e., the P_b_ center). These processes are: H^+^ approaches H atom of the Si-H bond; the Si-H bond length increases to form a H^+^-H-Si “bridge”; a H_2_ molecule and a positive defect D^+^ are formed.

For the first stage, proton can be released through reantants and reactions in Table 1.

#### 2.2.2. Hydrogen Diffusion Model

In 1985, Griscom [30] proposed the hydrogen diffusion model, which can be regarded as an improved model for hydrogen transport in the McLean model. Radiolytic molecular hydrogen is formed in the thermally grown SiO_2_ layer, and hydrogen diffusion determines the temperature and time dependence of the accumulation of interface states after irradiation.

In 1993, Conley et al. [31] observed that in thermally grown oxides exposed to molecular hydrogen at room temperature, interaction between E’ centers and hydrogen will occur. An E’ center involves an unpaired electron on a silicon backbonded to three oxygens. As the density of E’ centers decreases, D_it_ shows an increasing trend, and the saturation value of E’ change is within twice the saturation change of D_it_. This indicates that the E’/H_2_ reaction is likely to be involved in the interface trap formation process. Hydrogen may also be released from the bulk silicon in addition to the oxide layer.

#### 2.2.3. H_2_ Cracking Mechanism

In semiconductor manufacturing, molecular hydrogen (H_2_) is often used to form Si-H bonds at the Si/SiO_2_ interface, which can reduce interface trap density associated with dangling Si bonds. However, it has been found that excessive H_2_ in some radiation environments will increase the density of radiation-induced interface traps [32,33]. For example, Pease et al. [34] found that the performance degradation of flat-packaged linear bipolar temperature sensors was much greater than that of TO-52 can-packaged devices, because the molecular hydrogen content in the packaging environment of the former was considerably high. Chen et al. [19] also found that the bipolar junction test structure hermetically packaged with excessive molecular hydrogen exhibited enhanced degradation after radiation exposure.

The experiments and model by Stahlbush et al. [35] showed that H_2_ had a significant effect on interface; they also proposed a model to explain the formation of interface states. The dissociation of H_2_ may occur at two sites; one is the E’ center (i.e., the oxygen vacancy), and the other is the broken Si-O bond. The structure of the E’ center involves an unpaired electron on a silicon backbonded to three oxygens. H_2_ may undergo dissociation at a neutral or positively charged oxygen vacancy center. In 2008, Batyrev et al. [36] conducted experiments and simulations on H_2_ cracking in LPNP (Lateral PNP) BJTs. In this paper, a mechanism for the dissociation of hydrogen molecules and the generation of protons was proposed through first-principles calculations (1) A hydrogen molecule dissociates at the neutral oxygen vacancy and forms two Si-H complexes (≡Si-H, Equation (8)); (2) the Si-H complex captures a hole, then a hydrogen-bridge defect Si-H-Si (H_B_) is formed and a proton (H^+^) is released (Equation (9)).

H_2_ + VO → 2(≡Si-H) (8)

h + 2(≡Si-H) → H_B_ + H^+^ (9)

In 2012, Esqueda et al. [37] used a 1D model to simulate the effect of hydrogen on defect accumulation in dose rate effect. The results showed that the dissociation of molecular hydrogen at positively charged defects might be the key reaction. The dissociation of H_2_ is described by the following reactions, where D_C_ is hole trapping defect. H_2_ dissociates at the positively charged defect, and the products of the reaction are a DH center and a H^+^:

D_C_ + p → D_C_^+^ (10)

D_C_^+^ + H_2_ ⟷ D_C_H + H^+^ (11)

D_C_H + p → D_C_H^+^ (12)

D_C_H^+^ ⟷ D_C_ + H^+^ (13)

D_C_^+^ + n → D_C_ (14)

In 2000, Vitiello et al. [38] carried out DFT calculations on H_2_ dissociation. They found that at room temperature, H_2_ dissociation occurs only at a paramagnetic defect; at high temperatures, it occurs at diamagnetic centers.

Through chemical kinetics, a quantitative model was established to describe the relationship between excessive H_2_ and the formation of radiation-induced interface traps [19]. After hydrogen molecules reach the equilibrium concentration in the oxide, they react with neutral oxide defects and produce hydrogen-containing defects (DH) in the oxide bulk (Equation (15)); DH defects react with the holes to produce protons (Equation (16)).

H_2_ + 2D^+^ ⟷ 2DH (15)

h^+^ + DH → D + H^+^ (16)

In 2008, Fleetwood et al. [39] conducted several irradiation and hydrogen-soaking experiments on lateral and substrate PNP bipolar devices, which were irradiated in a ^60^Co source at a dose rate of 40 rad(SiO_2_)/s. It was found that with low dose rates and/or high hydrogen concentrations, hydrogen can react with oxygen-vacancy defects and change their structures, and holes are more likely to release H^+^ during transport. This is because the cross-section of H^+^ to capture electrons is several orders of magnitude smaller than that for slowly moving or metastable-trapped holes. This enhanced proton release at low dose rate or in oxide with a high hydrogen concentration leads to an increase in interface traps, which is the most common cause of enhanced gain degradation in lateral and substrate PNP bipolar transistors.

Hydrogen concentration in the oxide of bipolar transistor can be as high as 10^18^ cm^−3^ [40]. High concentration of hydrogen defects has a significant impact on proton generation rate and coefficient of electron–hole pair recombination. In 2009, Chen et al. [41] proposed a physical model to describe the dose rate and hydrogen effect in bipolar technology. Both electron–hole pair recombination and competing hydrogen reactions are considered in this model, in order to explain behaviors of bipolar devices and circuits at different dose rates. In this paper, the hole–hydrogen reaction is described by the continuity equation [42], and then simulated in a simulator called COMSOL finite element solver. Simulation results are in good agreement with the experimental results of Pease [42]. Among them, the protons responsible for the formation of interface traps are generated by the reaction of holes with hydrogen bond defects in the oxide.

(17) $$\frac{\partial H^{+}}{\partial t}=N_{\mathrm{DH}}\gamma_{\mathrm{DH}}p^{+}-[N_{\text{Si-H}}-N_{\mathrm{it}}(t)]\sigma_{\mathrm{it}}H^{+}-\frac{\partial f_{H^{+}}}{\partial x}$$

where H^+^ is the density of protons, γ_DH_ is the hole–hydrogen defect reaction rate constant, N_DH_ is the density of hydrogen defects in the oxide, N_it_ is the density of interface traps per unit area, N_Si−H_ is the density of passivated dangling bonds per unit area, σ_it_ is the reaction rate constant associated with the well-known interface trap generation process between protons and passivated dangling bonds at the Si/SiO_2_ interface, and f_H+_ is the proton flux.

In 2010, Tuttle et al. [43] studied the influence of the interaction between H_2_ and common defects in SiO_2_ on the accumulation of potential interface traps. H_2_ reacts with isolated dangling bonds and puckered vacancy defects.

In 2021, Liu et al. [44] conducted gamma-irradiation experiments on one type of GLPNP (Gated Lateral PNP) transistor at dose rates ranging from 0.167 mrad(SiO_2_)/s to 10 rad(SiO_2_)/s. It was found that the key mechanism for N_it_ enhancement in these devices is competition between the decomposition of hydrogen-related defects and their recombination with electrons. This paper analyzed H_2_ influence on interface trap formation, through experiments and first-principle calculations. First, H_2_ can dissociate on charged deep-level oxygen-vacancy traps near the interface, and releases protons. Secondly, H_2_ may also react with charged hydrogen-related defects (i.e., D_1_H^+^); this will further accelerate release of protons [44].

H_2_ + D_1_H^+^ → D_1_H_2_ + H^+^ (18)

In conclusion, H_2_ can diffuse into the oxide before or during irradiation; this diffusion will increase the probability of proton release. Subsequently, protons react at the Si/SiO_2_ interface to form interface traps. If a hole encounters a site that is favorable for proton release before being captured by a deep-level hole trap, the probability of interface trap generation will increase.

At present, the two main types of interface trap models are two-stage proton release model and the hydrogen dissociation model described above. (1) There are mainly two ways to generate protons: reaction of a hole with hydrogen species and hydrogen defect. (2) Hydrogen cracking mainly occurs at oxide defects, oxygen vacancy or broken silicon–oxygen bonds; among them, oxygen vacancy defect may be neutral or positively charged.

**Figure 3:**
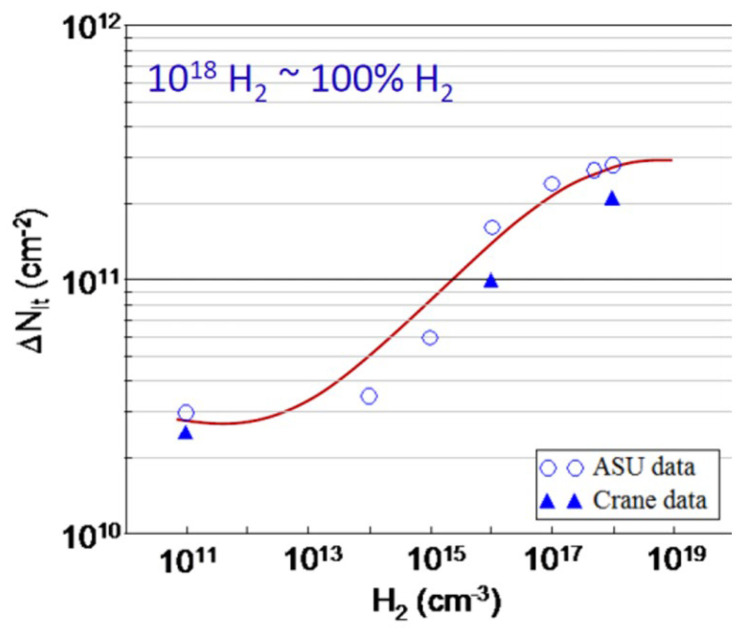
Interface trap formation due to excess H_2_ [46].

In 2011, Rowsey et al. [45] carried out TCAD (Technology Computer-Aided Design) simulations based on two models, explored key defects in the oxide that interact with radiation-induced charges to generate protons, and determined their contributions to ELDRS effect. As shown in Figure 3, it can be seen that at medium and high H_2_ concentrations, the hydrogen dissociation mechanism dominates [46]; these reactions are shown in the following. At low H_2_ concentrations, the direct release mechanism is predominant.

(19) $$V_{o\delta }^{+}+H_{2}\Leftrightarrow V_{o\delta }H+H^{+}$$

(20) $$V_{o\gamma }^{+}+H_{2}\Leftrightarrow V_{o\gamma }H+H^{+}$$

where $V_{o\delta }$ is a shallow hole trap, and $V_{o\gamma }$ is a deep hole trap.

## 3. Properties of Interface Traps

The properties of interface traps can influence the electrical performance of silicon-based devices. These properties mainly include defect concentration, defect types, charge states, energy levels, and capture cross-sections. Among these, the measurement of the interface trap concentration and its influencing factors is well established. This section focuses on reviewing the current research status of the types of interface traps and their electrical properties, specifically charge states, energy levels, and capture cross-sections, and attempts to analyze how these properties impact the electrical performance of silicon-based devices.

### 3.1. Types of Interface Traps

#### 3.1.1. Classification According to Physical Position and Energy Distribution

**Figure 4:**
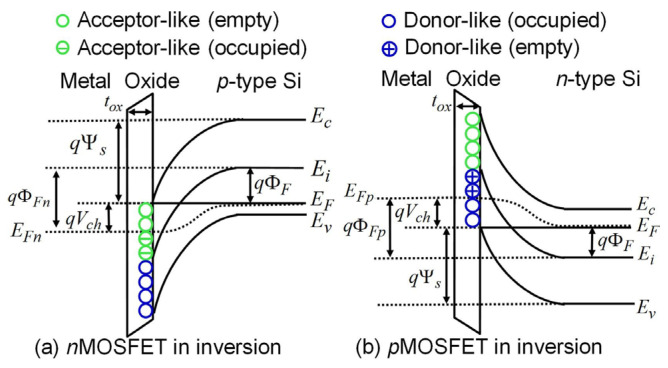
Energy band diagrams illustrating interface-charge trapping in (**a**) a bulk nMOSFET and (**b**) a bulk pMOSFET in inversion [48].

For interface traps, charge state is the most fundamental characteristic; it can be positive, neutral, or negative. Interface traps are therefore classified as donor and acceptor types. A donor interface trap is neutral when its level lies below the Fermi level (E_F_); if E_F_ moves above the Fermi level, it donates an electron and becomes positively charged. An acceptor interface trap is neutral when its level lies above E_F_; when electrons move below the Fermi level, the e trap captures an electron and becomes negatively charged (Figure 4). For example, when voltage is applied to the gate of a MOS device, the energy level of the interface trap moves upward or downward relative to the Fermi level. When the interface trap crosses the Fermi level, its charge state will change [47].

Early studies held that acceptor traps mainly reside above the intrinsic energy, and donor traps below it [40]. However, later work has shown different conclusions. Sah [49] analyzed changes in the C-V curves during the hole injection and annealing processes; he found that interface traps in the upper half of the bandgap are donor interface traps. The present consensus is that acceptor traps dominate the upper half of the Si bandgap, donor traps the lower half [50,51,52,53].

#### 3.1.2. Classification According to Defect Structure

**Figure 5:**
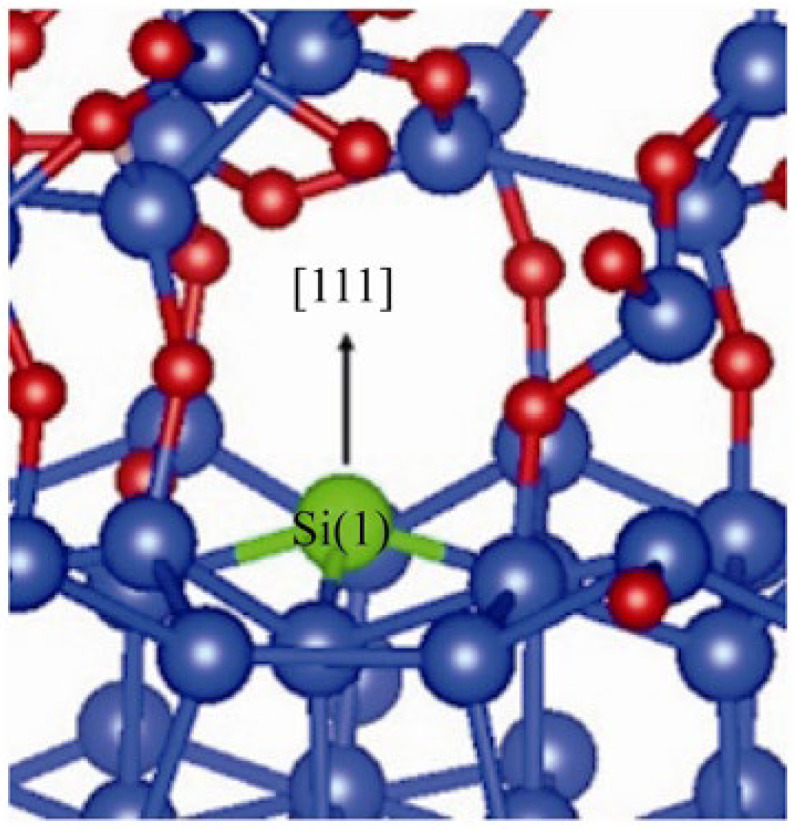
Structure of P_b_ defect which defect center atom Si(1) is marked in green [54].

Through ESR (Electron Paramagnetic Resonance) measurements, it was found that the P_b_ centers are the silicon dangling bonds at the Si/SiO_2_ interface. Ref. [54] compared the energy distribution of the P_b_ centers and the peak distribution of the interface traps, and found a good correspondence between them, which indicates that the P_b_ centers are the main source of the interface traps. In the early studies, it was believed that there was only one type of P_b_ center structure, see Figure 5.

**Figure 6:**
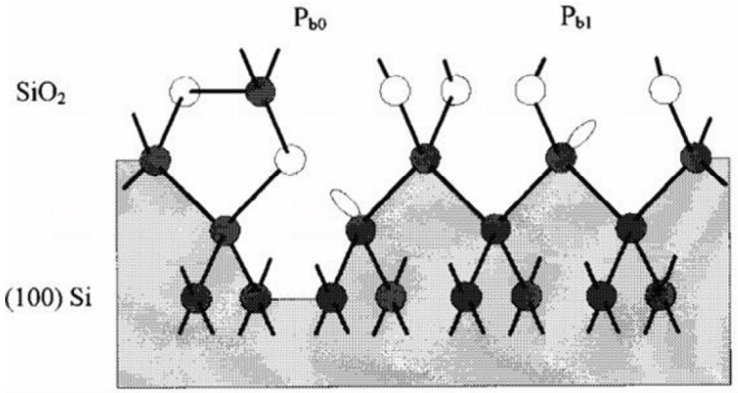
Structural model of the Si-SiO_2_ (100) interface [55].

With the development of measurement techniques, it is now generally believed that there are two types of P_b_ center structures, namely the P_b0_ and P_b1_ centers [47]. As shown in Figure 6 [55], the structure of P_b0_ is basically the same as that of the P_b_ center observed on Si/SiO_2_ (111), where they both have Si=Si_3_ structure; the structure of P_b1_ is different from the other two.

As can be seen from Figure 6, the P_b0_ (100) center has three nearest-neighbor silicon atoms and one second-nearest-neighbor oxygen atom, while the P_b1_ (100) center has one nearest-neighbor oxygen atom. Experimental results have demonstrated that the P_b0_ (100) center shares similar chemical properties with P_b_ (111).

### 3.2. Electrical Properties

In bipolar devices, the base current reaches its peak value when concentrations of electrons and holes are nearly equal; at this point, surface carrier recombination also reaches its maximum [56]. In order to characterize the electron activity of interface traps, energy levels (or spectra) and effective carrier cross-sections are usually used to evaluate their impacts on device performance. These trap energy levels serve as recombination centers, which will promote electron–hole recombination. The value of capture cross-section characterizes the capture efficiency of interface traps for carriers (hole or electron). The larger the capture cross-section, the higher the probability of capturing carriers, thus an increased recombination rate.

**Figure 7:**
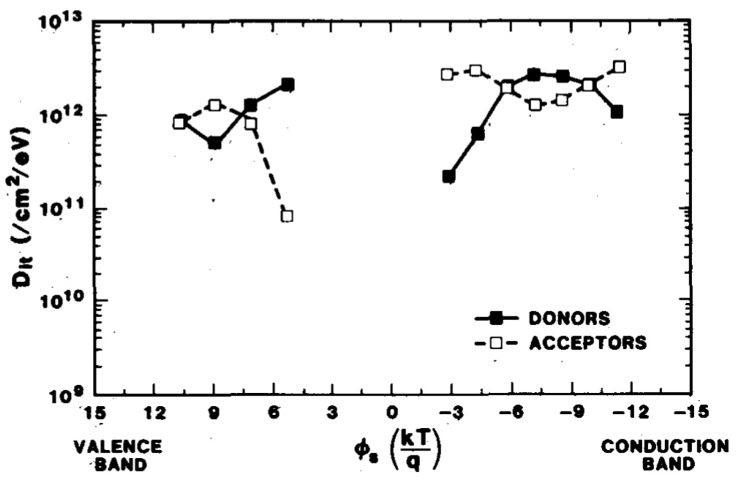
Distribution of donor and acceptor interface traps [57].

In 1988, McWhorter et al. determined the donor/acceptor nature of “radiation-induced” interface traps by combining quantitative equations with conductance measurement techniques [57]. As shown in Figure 7, the results indicate that both donor traps and acceptor traps exist in the two parts of the bandgap after irradiation, and their densities are relatively high.

**Figure 8:**
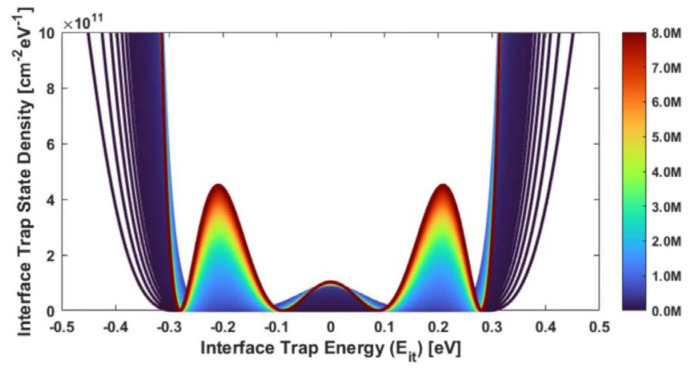
Density of interface trap states in Si/SiO_2_ across the energy band with respect to increasing TID [60].

The research results from Mishima et al. [58] in 2000 showed that the energy level of the P_b1_ center is close to the middle of the bandgap. In 2005, Chen et al. [59] measured the energy level distribution of interface traps in GLPNP and MOS devices, using GS (Gate Sweep) and SS (Sub-threshold Sweep) techniques. They found that the energy distribution of interface traps showed asymmetry. Compared with the energy below the mid-gap, the increase in N_it_ density is greater at energies above the mid-gap. Khoshnoud, A [60] also found that density of states across the energy band exibit a non-uniform manner (Figure 8).

### 3.3. Influence of Interface Trap Properties on Device Performance

#### 3.3.1. Influence of Interface Trap Concentration

An increase in interface trap concentration raises the surface recombination velocity (SRV), thereby causing an increase in peak current amplitude, i.e., enlarging the recombination current peak. Therefore, the decrease in the current gain can be suppressed by reducing the carrier recombination rate [61,62]. For example, in the case of low-level injection, the relationship between SRV and N_it_ can be described by the following equations.

(21) $$\mathrm{SRV}≅{\sigma N}_{\mathrm{it}}\nu_{\mathrm{th}}$$

wherein σ is the trap capture cross-section, and ν_th_ is the thermal velocity of the carriers.

(22) $$\Delta I_{B}\approx q\sigma \nu_{t}\Delta N_{\mathrm{it}}\frac{n_{s}{(\psi }_{s}{)p}_{s}{(\psi }_{s}{)-n}_{i}^{2}}{n_{s}{(\psi }_{s}{)+p}_{s}{(\psi }_{s}{)+2n}_{i}}S$$

where q is the electronic charge, n_s_ and p_s_ are carrier concentrations at the surface, ψ_s_ is the surface potential, and S is the surface area over which recombination takes place. It can be seen from the above equations that an increase in N_it_ will lead to an increase in the surface recombination rate, thus causing an increase in the base current.

Zhuang Yiqi and co-workers [63] studied how interface traps at SiO_2_-Si interface affect current amplification factor (h_FE_) of bipolar transistors; a change in charge amount on the interface traps will cause a change in the base ionization through the base region surface potential, ultimately leading to h_FE_ drift. Experiments and models showed that h_FE_ drift is proportional to the interface trap density N_it_.

#### 3.3.2. Influence of Interface Trap Energy Levels and Capture Cross-Sections

Energy levels of interface traps have significant impacts on the electrical performance of bipolar devices, mainly manifested as reducing the channel mobility, affecting the threshold voltage, increasing the on-state voltage drop and breakdown voltage, increasing the leakage current, etc.

**Figure 9:**
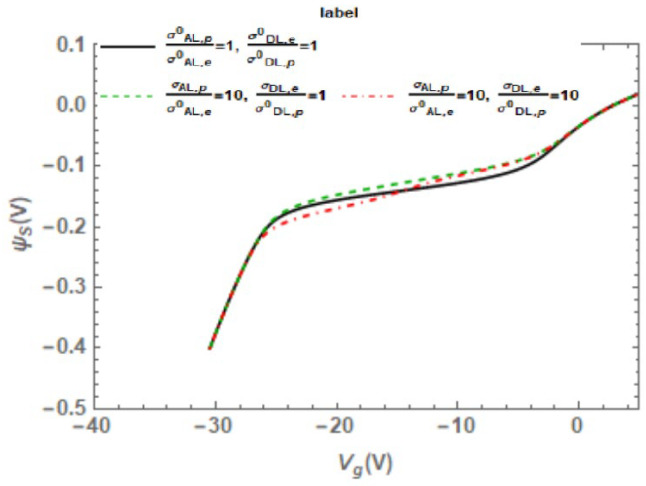
The dependence of the surface potential on cross-sections.

The electron/hole capture cross-section of interface traps will affect the carrier-recombination rate. Duan [64] explains their relationship. As Figure 9 illustrates, the cross-section has a negligible effect on surface potential. The relationship between the base current and surface recombination caused by donor-like/acceptor-like interface traps is shown in Equation (23); thus, when the intrinsic energy level is close to the hole quasi-Fermi level or electron quasi-Fermi level, base current reaches its peak.

(23) $$\Delta I_{B}\approx \mathrm{qS}\;(\Delta {\mathrm{SRV}}_{\left(\mathrm{DL}\right)}+\Delta {\mathrm{SRV}}_{\left(\mathrm{AL}\right)})$$

In 1990, Shaneyfelt et al. [27] conducted experiments on MOS devices and found that under high electric field conditions, the effective hole capture cross-section in SiO_2_ decreased and the number of interface traps reduced, which had an E^−1/2^ relationship with electric field.

#### 3.3.3. Influence of Interface Trap Charge State

**Figure 10:**
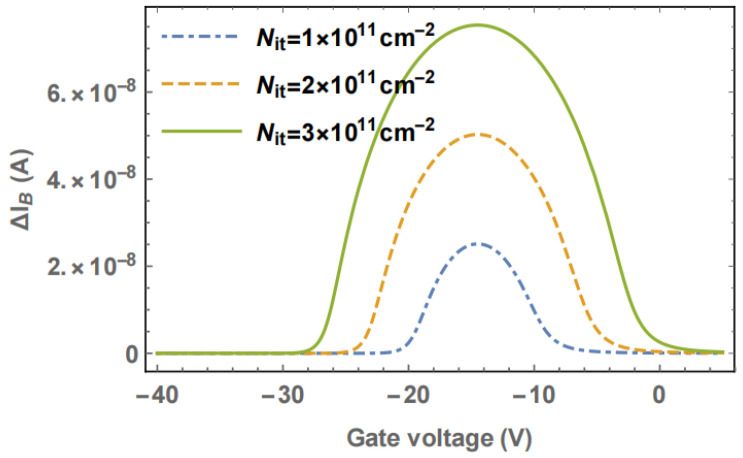
Effect of interface trap charge on peak broadening.

In 2004, Chen et al. [65] from the University of Arizona found that the GLPNP showed a broadened peak distribution when irradiated with ^60^Co at a dose rate of 39 rad/s, in addition to an increase in the peak value of the base current. As shown in Figure 10, with the increase in the interface trap density, both the peak base current and the width of the peak increase. This is due to the change in interface trap charge state, which lies in the oxide above the base region.

Ref. [65] showed that the energy distribution of interface traps has a significant impact on the curve shape of the base current versus the gate voltage. (1) If acceptor and donor interface traps are symmetrically distributed around mid-gap with equal densities, no peak broadening or shift is observed near mid-gap; at other energy level positions, it leads to a decrease in the peak current. (2) In an asymmetric distribution, with acceptor interface traps far from the mid-gap, recombination efficiency weakens. This will generate the tilting of the current peak and a decrease in its peak value.

In 2007, Chen et al. [62] conducted further studies on the broadening of the peak base current distribution. When gate bias sweeps from accumulation to inversion across the base, the charge state of the interface traps will change. The interface traps electrostatically affect the carrier concentration, thereby influencing its base current. This paper analyzed the theoretical relationship between the gate voltage and the surface potential (Equation (24)) and calculated the charge state of the interface traps by determining the energy of the interface traps relative to the effective Fermi level. It can be seen that the broadening of the peak base current observed in the experiment is caused by the change in the charge state of the interface traps.

(24) $$V_{g}=V_{\mathrm{fb}}{(\psi }_{s}{)+\psi }_{s}-\frac{Q_{s}{(\psi }_{s})}{C_{\mathrm{ox}}}$$

where Q_s_ is the total charge per unit area induced in the silicon, V_fb_ is the flat band voltage, C_ox_ is the capacitance per unit area between the gate and active base region, V_g_ is gate voltage, ψ_s_ is the surface potential.

**Figure 11:**
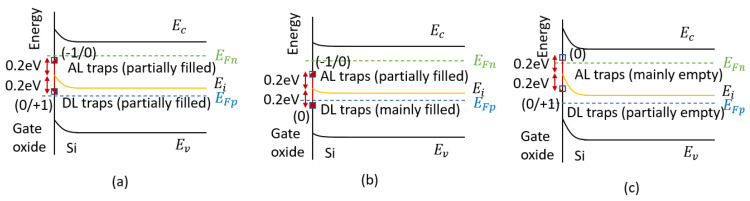
Schematic illustration of the band structure and charge status of traps below the gate oxide. (**a**) σ_e_ = σ_h_. (**b**) σ_e_ ≠ σ_h_, smaller negative value of V_G_. (**c**) σ_e_ ≠ σ_h_, larger negative value of V_G_.

In 2024, Duan et al. [64] from the China Academy of Engineering Physics conducted low-dose-rate radiation experiments on GLPNP transistors. Both the experimental and calculated results show that one of the reasons for the splitting of the base current peak is the difference in electron cross-section (σ_e_) and hole cross-section (σ_h_) of acceptor- and donor-like defects formed near SiO_2_/Si interface. When σ_e_ is the same as σ_h_, the base current reaches its maximum value, and the intrinsic energy level of silicon is located at the center of the quasi-Fermi energies of electrons and holes, as shown in Figure 11a. The two current peaks correspond to the cases when the intrinsic energy level is close to the hole quasi-Fermi level (for acceptor traps, Figure 11b) or the electron quasi-Fermi level (for donor traps, Figure 11c).

## 4. Influencing Factors of Interface Trap Formation

The formation of interface traps in silicon-based devices is a complex process, and its influencing factors cover various aspects such as material properties, manufacturing processes, environments, and operating conditions. Here we summarize the impacts of electric field, temperature, bias conditions, and hydrogen (H_2_) concentration on interface traps under a low-dose-rate irradiation environment.

### 4.1. Electric Field/Bias

**Figure 12:**
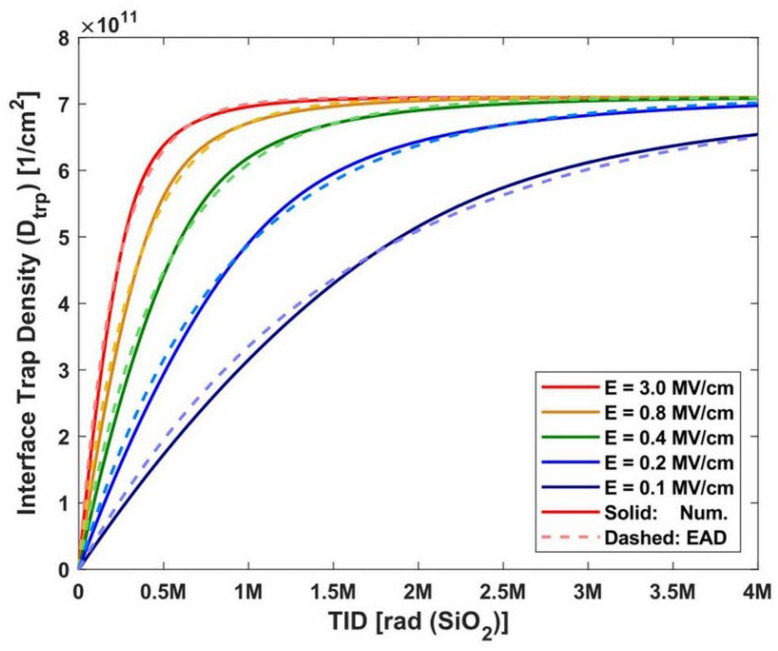
EAD function results in comparison with numerical results under different electric field conditions [60].

In 1977, Winokur et al. [66] studied the dependence of interface states on electric field and time. Figure 12 shows that the interface trap density increases with the positive electric field, indicating a strong field dependence.

In 1990, the experiment by Shaneyfelt et al. [27] showed that there is a quantitative relationship of E^−1/2^ between the interface state density and the electric field. This relationship appears both under a positive and a negative electric field after annealing with a positive bias voltage. An experiment was performed under gamma radiation with a 49 mrad(SiO_2_)/s dose rate. In contrast, Schwank et al. [67] found a decrease in the interface trap density at a high gate bias on n-type substrate capacitors.

In 1991, Brown et al. [68] from the Naval Research Laboratory in Washington, in their experiment on the interface traps of MOS devices, found that the formation time of interface traps strongly depends on the electric field of the oxide layer. When the field intensity of the oxide layer increases by a factor of 8, the formation time of interface traps decreases by about 20 times.

In 2006, Chen et al. [65] from Arizona State University conducted an annealing experiment on GLPNP devices. They found that when irradiated at 0 V, most devices’ interface traps decrease with the decrease in annealing time and temperature. However, for devices irradiated at −50 V, the interface trap density increases.

Ref. [66] showed that in wet-oxide n-type MOS devices, more holes reach the interface under positive gate bias, leading to a higher interface-trap density. Ref. [21] presents an opposite trend; this is due to the field dependence of the hole capture cross-section at the Si/SiO_2_ interface, whereas in Ref. [65], the increase in interface trap density for devices under −50 V bias stems from different annealing mechanisms for border traps and interface traps.

### 4.2. H_2_ Concentration

Hydrogen is one of the dominant factors determining the total dose and dose-rate characteristics of linear bipolar circuits. The percentage of hydrogen increases the degree of performance degradation at low dose rates.

**Figure 13:**
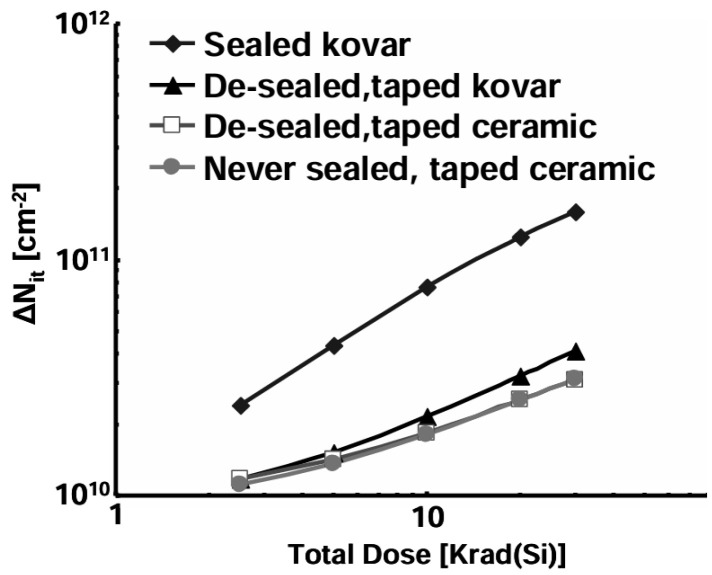
Radiation induced interface trap build-up in devices with unsealed and hermetically sealed packages [46].

**Figure 14:**
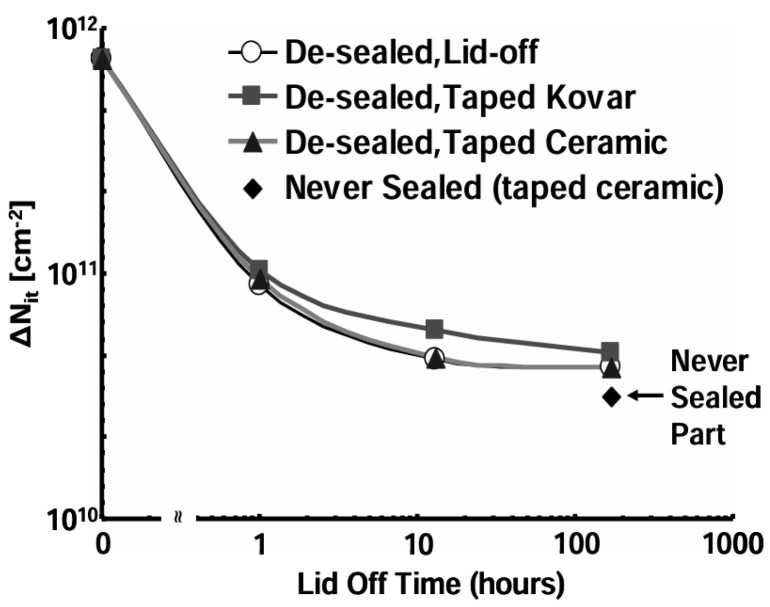
Decrease of radiation induced interface traps as H_2_ diffuses out of the sealed package over time [46].

Schwank from Sandia National Laboratories studied hydrogen’s role in MOS polysilicon-gate capacitors [67]. It was found that if high-temperature annealing is carried out in hydrogen, the concentrations of both interface traps and oxide trapped charge measured immediately after irradiation will increase, measured immediately after irradiation. Experiment results in [46] showed the same trend, see Figure 13: sample with hermetically sealed (1% in package H_2_) exhibited the worst degradation; Figure 14 shows the decrease in radiation damage as H_2_ is diffused out of the package. The increase in the amount of hydrogen used is related to the early rapid accumulation of interface traps (from 1 ms to 10 s) and affects the total number of interface traps generated.

**Figure 15:**
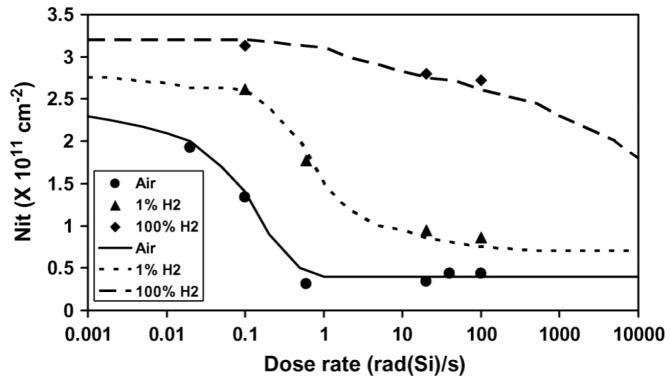
ΔN_it_ as a function of dose rate after irradiation in three different ambient hydrogen conditions [69].

In Ref. [19] published by Chen et al., experimental results of interface traps in environments with various hydrogen concentrations were presented, and the hydrogen concentrations were 0.01%, 0.1%, 1%, 10%, 50%, and 100%, respectively. Their 2009 data showed that H_2_ concentration has an important influence on the interface trap density. Figure 15 shows the dose-rate effect of GLPNP bipolar devices under different hydrogen concentrations. It can be found that the limit of the low dose rate shows an upward trend with the increase in the hydrogen concentration. The main reason for this trend is the competition between electron–hole recombination and hole–hydrogen reaction.

Adell et al. carried out a hydrogen immersion experiment on bipolar devices [70]. The results showed that 0.1% was the limit of H_2_ concentration affecting device degradation.

In 2018, Li et al. from Harbin Institute of Technology [71] found through experiments on transistors immersed in H_2_ that the degradation was much greater than that of devices without H_2_ immersion. This is because the ionization-induced release, transport, and reaction of hydrogen in the bipolar base oxide greatly enhanced the formation of interface traps.

Yang et al. [72] from Harbin Institute of Technology studied the influence of 3 MeV proton irradiation on GLPNP transistors, including two environments: with and without hydrogen molecule H_2_ immersion. The 3 MeV protons mainly produce ionization damage in GLPNP transistors, and hydrogen will exacerbate the formation of interface traps in GLPNP transistors, leading to more severe damage to GLPNP transistors.

### 4.3. Temperature

In 1995, Schrimpf et al. from the University of Arizona, when conducting high-temperature annealing experiments [73] according to the MIL-STD-883B method, found that as the temperature became higher, the device performance degradation increased significantly. The formation of interface traps will be accelerated while annealing at 100 °C. Boch et al. [74] explored the temperature dependence in linear bipolar devices; excess base current gradually increased with the increasing temperature, then decreased at higher temperatures, after reaching its saturation value.

The experimental results of Pershenkov et al. in 2007 [75] showed that when transistors were irradiated by 40 kV Cu-anode X-ray 2 krad(SiO_2_)/s, high-temperature irradiation would lead to an increase in the accumulation of interface traps (a high temperature of 100 °C was used in the experiment), and this phenomenon occurred under three bias conditions: forward bias, zero bias, and reverse bias.

ETI (elevated temperature irradiation) is used to predict the device performance degradation at a higher dose rate for a low dose rate. At a given dose rate, the radiation-induced interface trap concentration depends on the irradiation temperature and the total dose. In 1996, Witczak et al. [76] found that raising the ETI temperature enlarged the excess base current of LPNP transistor, and more interface traps were created. However, at a higher irradiation temperature, interface traps saturate and then decrease. In 2011, David R. Hughart et al. [77] from Vanderbilt University studied the formation and annealing mechanisms of interface traps in the ETI environment. It was found that with the increase in the radiation temperature and the total dose, the accumulation of interface traps first increased and then decreased, reflecting the competing passivation/depassivation reactions at the interface. This is consistent with the experimental results of Witczak.

**Figure 16:**
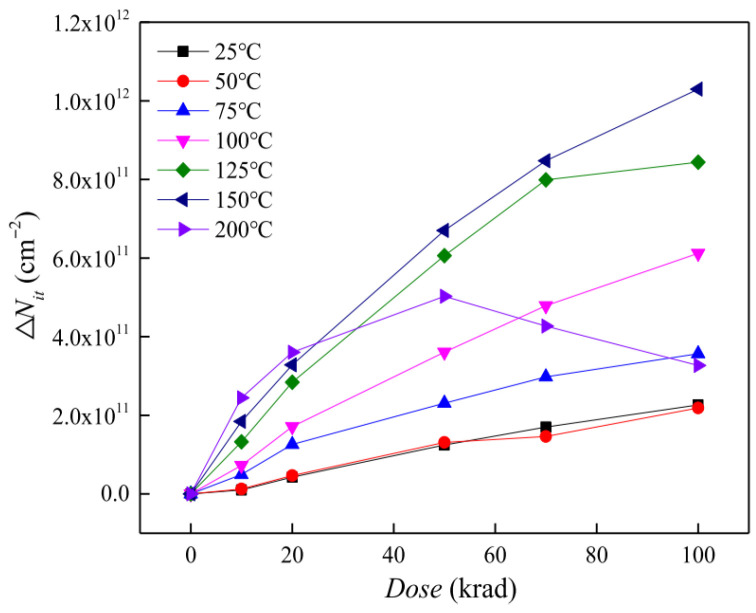
Estimated radiation-induced interface trap buildup versus dose in the oxide for the GLPNP transistors irradiated at different temperatures [69].

DLTS (deep-level transient spectroscopy) can be used to analyze the formation of the ionization defects in bipolar transistors at different temperatures; an increase in interface traps will occur with the increase in temperature (Figure 15). Dong et al. [78] from Harbin Institute of Technology studied the evolution of ionization radiation-induced defects in GLPNP bipolar transistors at different temperatures, with ^60^Co gamma irradiation at 10 rad (Si)/s. Both GS curve and DLTS measurements confirmed that high temperatures would accelerate the ionization damage. As shown in Figure 16, when the irradiation dose is less than 25 krad, N_it_ increases as the temperature gradually rises at a given dose. However, when dose exceeds 25 krad, N_it_ first increases and then begins to decrease at 200 °C.

**Figure 17:**
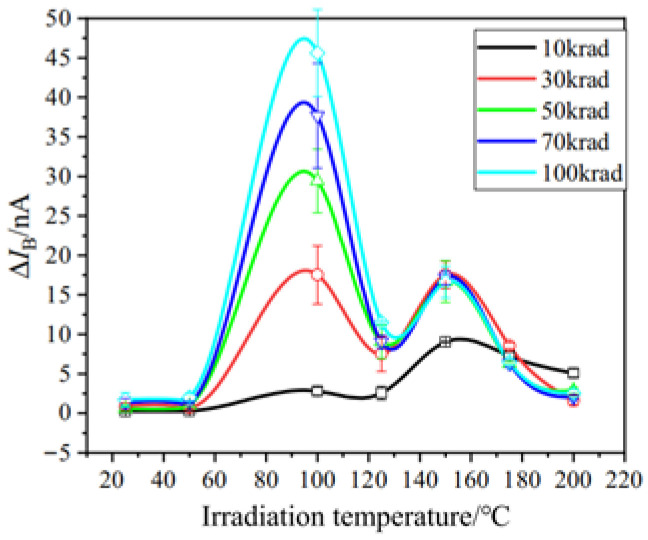
The optimal temperature at different doses [69].

In 2023, Hang Zhou et al. from the China Academy of Engineering Physics [79] studied the defect formation induced by ionizing radiation in GLPNP bipolar transistors at different temperatures. Figure 17 shows the excess base current versus the irradiation temperature. It can be inferred that there are two temperatures that can effectively promote interface trap buildup at the SiO_2_/Si interface: 100 °C and 150 °C, respectively. This double optimal temperature is attributed to the competing processes of oxide-trap conversion into interface traps and concurrent interface-trap annealing.

Based on the above observations, we can conclude that at high temperatures (150–200 °C), oxygen vacancies (e.g., VH centers) react with protons at Si/SiO_2_ interface, reducing the proton supply required for interface trap formation, thereby leading to the “rise-then-fall” trend in interface trap density. Secondly, the appearance of a second peak in the interface trap concentration may be caused by the conversion of oxide trap charges into additional interface trap charges.

## 5. Challenges and Future Outlook

Silicon-based devices will experience performance degradation under an irradiation environment, especially at low dose rates, where the formation of interface traps is the dominant mechanism. These interface traps exhibit different structures and electrical properties, e.g., asymmetric energy level distributions and capture cross-sections, that influence the degradation in different ways. Recent work has also pointed out the role of border traps (near-interface traps). Based on the above research, challenges and possible research directions are proposed.

(1) The main types of interface defects have been identified, but there is still no definite conclusion on the degree to which different types of interface defects affect the performance of silicon-based devices, for example, the role played by donor- and acceptor-type interface traps in device performance degradation.
(2) The energy levels of interface defects are different, and they also have different capture cross-sections for electrons/holes, which will affect carrier recombination rate and thus the electrical performance of the devices. Therefore, it is necessary to conduct further microscopic research on the electrical properties of interface traps.
(3) Devices with different structures exhibit different variation curves of interface traps under environments such as temperature and electric field. It is necessary to study the mechanisms causing these phenomena.
(4) It needs more study on the properties of near-interface traps, and their impacts on the electrical performance of devices.

Further research and analysis of the above issues will provide a powerful basis for the anti-radiation hardening technology of devices and improve the reliability of devices in a radiation environment.

## Figures and Tables

**Table 1:** Reactants and eactions for proton formation.

Reactant 1	Reactant 2	Reaction(s)
hole	H^0^/H_2_	Equation (3)
hole	Hydrogenated defects	Equations (4) and (5)

## Data Availability

No new data were created or analyzed in this study.
